# Development and Innovation in Cooked Ham Produced in Spain

**DOI:** 10.3390/foods12071360

**Published:** 2023-03-23

**Authors:** Cristian B. Arenas, Beatriz García-Béjar, Ana Santos, Almudena Soriano

**Affiliations:** 1 Department of Inorganic Chemistry, Faculty of Chemical Sciences, Complutense University of Madrid, 28040 Madrid, Spain; 2 Area of Food Technology, Faculty of Chemical Sciences and Technologies, University of Castilla-La Mancha, Avda. Camilo José Cela, 10, 13071 Ciudad Real, Spain; 3 Regional Institute for Applied Scientific Research (IRICA), University of Castilla-La Mancha, Avda. Camilo José Cela 10, 13071 Ciudad Real, Spain

**Keywords:** cooked ham, development and innovation, production process, ingredients and additives, nutritional and health claims

## Abstract

The production of cooked ham has been gaining popularity in recent years in Spain. In general, the production process carried out by the companies remains traditional, and different production methods are therefore being sought to innovate and improve the quality of the product. This is either through pig crossbreeding, varying additives and ingredients, improving some stages of the production process, or providing nutritional and health claims that are useful to guiding the purchasing decision of consumers. Obviously, this series of changes must be subject to Spanish and European regulations in order to be marketed inside and outside the country.

## 1. Introduction

Spain is the second largest producer of pork meat in Europe, narrowly surpassed only by Germany, and ranks eighth in the world, below countries such as China, the United States and Brazil [1]. A highly prestigious meat product derived from pork that is widely consumed is the cooked ham; this is fundamentally in the “extra” category, perfectly stipulated by the current Quality Standard for Meat Derivatives in Spain (Real Decreto 474/2014). However, the production process of cooked ham remains very traditional, so any innovation in the process in the search for an improvement in the product’s quality arouses great interest. Currently, several leading Spanish meat industry companies are committed to researching, developing and innovating this product, given its importance in the national and international markets. It is therefore necessary to review the current state of the development of this product and any other underlying aspects, such as the most suitable pig crossbreed for use in its production, brine components, stages of the production process, labelling, etc., all aspects in which innovation is possible.

## 2. Definition of Cooked Ham

The current Quality Standard for Meat Derivatives in Spain (Real Decreto 474/2014) defines cooked ham as a pasteurised meat product obtained from anatomically identifiable pieces of meat or pieces thereof, in which the muscle bundles are recognizable. These cuts are subjected to brining, massaging, resting and a heat treatment in order to achieve, in its internal part, a partial or total coagulation of its proteins. Optionally, a moulding step is included before heating to give the product the appropriate shape. Two commercial categories of cooked ham can be found on the national market, this categorisation deriving mainly from the meat content and type of additives. The cooked ham category denoted “extra” usually has a meat content higher than 80%, while cooked ham without any commercial category designation (considering as standard quality) is that with a meat content between 70–80%. If starch is included as an ingredient in the product, the designation will change to “fiambre” and will be cheaper in the market. Table 1 specifies the characteristics that the “fiambre” of cooked ham and cooked ham must have to belong to any category (Real Decreto 474/2014).

However, in the future, it may be necessary to establish new parameters for the categorisation of cooked ham as there has been a downward trend in the number of ingredients/additives used in the product in order to meet new consumer demands for more “natural products”. In recent years, from 2016 to 2020, the average sugar content has changed from 1.50 g/100 g to 1.35 g/100 g, and average salt from 2.30 g/100 g to 1.93 g/100 g [2].

## 3. Innovation and Development in the Choice of Raw Materials

It is difficult to determine which breed or crossbreed of pig, as selected by breeding enterprises, is the most suitable for obtaining the highest quality of cooked ham. Traditionally, white pigs have been used in Spain, including crossbreeds of Landrace, Large White or Pietrain, because of their high-quality production and meat yield, as well as the resulting quality of the finished product. In recent years, the popularity and consumer recognition of the Duroc pig breed has increased. Pigs of this breed originate from the USA, are characterised by a reddish pigmented coat, are rustic and easily adapt to the environment. For this reason, its breeding is widespread worldwide and it is appreciated for the high-quality products it provides. Duroc provides meat pieces with a relatively high fat infiltration in the muscle and a high resistance to stress, showing a low incidence of the technological defect PSE (pale, soft and exudative meat) [3].

One of the most interesting qualities offered by Duroc is its possibility of being crossed with an indigenous species, such as the Iberian pig. Spanish legislation states that the crossbreeding of a Duroc male and an Iberian female gives a litter that is catalogued as 50% Iberian pig (Real Decreto 4/2014). However, a lack of information about the use of the Duroc or Iberian breed as a raw material for the production of cooked ham has been observed. Therefore, a study in this sense could be interesting and valuable. The main difficulty that both breeds present is their high amount of intramuscular fat, especially in the Iberian; this would generate a cooked ham with too much marbling and flavour. This might not be acceptable or suitable for the consumer, since a leaner product is expected. On the other hand, whether the use of these pigs could be economically profitable should be studied. Nevertheless, all raw material that aims to obtain the denomination of “cooked ham” must first comply with the Quality Standards (Real Decreto 474/2014), regardless of the type of pig it comes from.

## 4. Innovation and Development in the Elaboration Process

Cooked ham is produced in a traditional and standardised manner in the meat industry in the following stages:

1. Reception of raw hams. Raw hams are usually received chilled or frozen at 3–4 °C or at −18 °C, respectively. In the case of the production of high-quality cooked ham, prior freezing of the raw material is discouraged because it negatively affects the quality of the final product. Freezing causes the following problems: protein oxidation, the loss of red colouring and hardness [4].
2. Polishing. The polishing of the hind leg consists of the complete removal of all bones, tendons, and all or part of the skin and the subcutaneous fat, keeping the muscle bundles intact. The level of polishing of the raw material is according to the quality or category of the final product [5] and is carried out by specialised staff.
3. Weighing of the piece. This stage is important so that the percentage of brine to be injected in the subsequent stage is known. It is usually carried out automatically, in line with the weighing system.
4. Brine injection. Currently, the most used form of brine injection is a multi-needle system. It is carried out at temperatures between 3 and 8 °C, since an increase in temperature promotes the penetration of the salt but reduces the microbiological stability of the piece [5]. It is advisable to replace the brine every 24 h to prevent the loss of antioxidants and nitrites due to the brine’s high reactivity, and to prevent microbial growth. Multi-needle injection systems allow the homogenisation of the brine and are grouped into two types: low pressure and spray effect. Spray effect systems have the particularity of being able to dose the volume of brine with a spray or atomiser effect. In other words, once the needles have completed their journey through the piece of meat, the brine is injected with a spray effect at high pressure, between 6 kg/cm^2^ and 10 kg/cm^2^, guaranteeing homogeneity [6]. In addition to the injection system, the percentage of brine injected can be optimised. The most commonly used ranges from an industrial point of view are very wide, between 25 and 40% of the piece weight [7], affecting the quality of the final product.
5. Tumbling massaging or malaxing. The main objective of this step is to distribute the brine and solubilise the meat proteins that facilitate the binding of the product. It is performed in rotating cylinders for several hours depending on the amount of brine injected, interspersed with rotation and rest phases at temperatures of 3–6 °C under vacuum to avoid foam formation during the process [8].
6. Maturation of meat. The introduction of this stage is recommended in the production of high-quality cooked ham because it influences the organoleptic characteristics, mainly texture. In this stage, the protein extraction carried out during malaxing will be completed and higher levels of water retention, muscle bonding, and a homogeneous colour will be also achieved [6]. Maturation consists of keeping the ham at refrigeration temperatures for normally between 12 and 18 h, sometimes up to 48 h. The inclusion of this stage in the process influences its cost-effectiveness [6].
7. Moulding. The ham is placed in a cotton mesh or plastic bag, and then in stainless steel moulds that give it the characteristic shape to be sold on the market. They are usually oval, rectangular or mandolin shaped. They can also be placed in heat-shrinkable bags, which retract as the ham does, forming the final packaging in which it will be sold.
8. Pressing and/or resting. The cooking mould is closed and at the same time, is pressed and left to rest in order to ensure the absence of any remaining air, whether occluded in the mass or that which may remain between the product and the packaging material.
9. Cooking. This is a delicate stage of the process that requires good control. It is important to reach pasteurisation temperature values in the centre of the piece that are usually between 65 and 72 °C [9]. The aim of this is the destruction of the viable cells of microorganisms and producing enzymatic inactivation, without altering the sensory characteristics of the ham. The microorganisms naturally present in ham are various (lactobacilli, staphylococci, enterococci and micrococci), and the treatment conditions are set according to the most heat-resistant microorganisms (enterococci) [10]. On the other hand, the minimum temperature to achieve the destruction of *Trichinella spiralis* is stipulated as being 58.3 °C [9].

In addition, cooking also aims to achieve a firm consistency, the correct cohesion and tenderness of the product, which occurs after the coagulation of the proteins and partial dehydration, as well as the desired pink colour that occurs due to the denaturation of the nitrosomyoglobin [11]. Cooking is usually carried out in hot water baths and is recommended, as two mechanisms of heat transport are involved: convection (heat is directed from the medium to the surface of the ham) and conduction (heat is transferred from the outside of the ham to the inside) [12]. Heat transfer can also be performed with hot air, but it gives a worse yield and lesser product cohesion [5]. There are several traditional cooking methods [13]: (i) at a fixed temperature, with the disadvantage that the temperature inside the ham must be perfectly controlled; (ii) cooking until the desired temperature is reached inside but the surface of the ham is overexposed; and (iii) step cooking with the following bath temperatures: 1 h at 40 °C, 1 h at 50 °C, 1 h at 60 °C and the rest completed at 78 °C. Slow cooking has been shown to improve product cohesion, tenderness and reduce cooking damage [14]. An alternative is vacuum cooking or *sous vide* cooking, which is performed by packaging the ham in airtight, heat-stable, hermetically sealed plastic and then applying a vacuum and a moderate cooking temperature (65–90 °C in the centre of the product) for a long time (2–8 h). In short, this is an LT–LT (Low Temperature–Long Time) treatment [15].

Then, the temperature must be quickly lowered (to 0–3 °C in the centre of the product) and kept in refrigeration. The absence of oxygen inside the bag extends the shelf life of the product, as it hinders the growth of aerobic microorganisms, as well as the development of lipid oxidation. Therefore, and in relation to shelf life, the *sous vide* method also avoids the generation of off-flavours prematurely by controlling both aspects (microbial growth and lipid oxidation) [15].

Finally, it should be considered that, for cooked ham, the temperature should not exceed 65 °C, since above this value, the solubilisation of the collagen occurs, with the consequent formation of gelatine and the loss of meat toughness. Below this temperature, a greater coagulation of proteins is avoided and, consequently, there is a reduced toughness after cooking, which is important for the texture of the meat [16].

10. Cooling. Pasteurisation eliminates the viable cells of microorganisms, but it does not eliminate spores from spore-forming bacteria, which can germinate if immediate chilling (4–5 °C) is not carried out after cooking in order to ensure the product’s stability before consumption. This process can be performed by using air shocks, immersion or water showers. The decrease in the temperature is the most crucial part and should be limited to less than 4 h [13,17].
11. Final stages. The cooked ham is unmoulded, sliced, packaged and finally stored and distributed. Whole pieces are vacuum packed. A squirt of gelatine is usually introduced into the bag before sealing, which uses a vacuum in order to cover gaps and to homogenise the appearance. When the cooked ham is sliced, modified atmospheres without oxygen (normally a mixture of N_2_/CO_2_) are used for packaging. Slicing can only be performed in clean rooms with extremely hygienic air quality. Market storage is between 2 and 4 °C.

## 5. Innovation and Development in the Use of Ingredients and Additives

The most common ingredients and additives used in cooked ham are presented below, as well as their functions. The additives authorised for cooked ham are clearly set out in Europe by Regulation (EU) No. 1129/2011.

### 5.1. Ingredients

1. Water. The added water is used to dissolve the rest of the ingredients and additives injected in the form of brine. After meat, it is the second most abundant component in a cooked ham, and it is desirable that the water used as a brine matrix is weakly mineralised. The presence of metal ions can affect other additives; for example, traces of iron or copper can partially destroy ascorbate, which destabilises the colour of the final product, and also poses a toxicological risk [18].
2. Common salt. Common salt or sodium chloride (NaCl) provides a salty taste, firmness, aroma (by interaction with other components) and a reduction in water activity, inhibiting the growth of microorganisms and enabling preservation [19]. It also contributes in other ways, as it possesses the ability to increase the water holding capacity (WHC) of the ham at an alkaline pH through the solubilisation of meat proteins, resulting in increased binding between muscles. This is due to the ionic strength of the salt, which is able to weaken the electrostatic bonds between amino (NH_4_^+^) and carboxyl (COO^−^) groups in the quaternary structure of proteins [20,21]. In addition, it promotes lipid oxidation. Although the mechanism is not fully elucidated, it is quite possible that the initiation of lipid oxidation is due to the formation of radicals by high hydrostatic pressure [22]. Studies point to several pro-oxidative mechanisms of NaCl, indicating that it is due to the following: (i) its ability to penetrate cell membranes that facilitate the entry of oxidising agents; (ii) the release of iron ions from haemoprotein molecules; and (iii) the inhibition of antioxidant enzymes such as catalase, protease, peroxidase and superoxide dismutase [22]. In low-salt cooked ham, part of NaCl is often replaced by KCl and the taste of the product is slightly affected [23].
3. Sugars. Although their use is not essential, they can be added to cooked ham. However, like salt, the amount used by the industry continues to decrease over the years, according to the recommendations of the WHO and the European Commission; these are in line with the NAOS Strategy (2005), which has been working with different sectors to reduce the levels of salt, sugar, fat and calories in food products. Sugars are used in cooked ham for two reasons: (i) to increase the bacteriostatic power by decreasing the activity of water and (ii) to improve the flavour of the ham by providing some sweetness [2,24]. Saccharose and dextrose are the most common sugars in the cooked ham industry. Saccharose contributes to the taste of the finished product, but its ability to reduce water activity is limited by its sweetening power, as limit values of only around 0.8–0.9% can be achieved. However, the most suitable concentration would be around 0.5%. Dextrose has a higher sweetening capacity than saccharose and a higher osmotic pressure; in finished products, 3% can be reached in the brine without affecting the taste too much. The main problem is that, as it is a monosaccharide, it is digested more quickly by microorganisms [18]. Resconi et al. [25] proposed the use of fructo-olysaccharides as a healthier substitute for dextrose in cooked ham, concluding that there was no significant sensory difference. Therefore, it might be suggested as an alternative to reduce sugar content.

### 5.2. Additives

The most common additives found in the cooked ham industry are classified as preservatives, stabilisers and antioxidants:

1. Preservatives. These are a series of chemical substances that are used to minimise the deterioration caused by microorganisms (mainly moulds and bacteria), avoiding economic losses to a company. Improvements in the cold chain and in the production stages, together with strict legislation and public opinion against their use, are leading to a reduction in the use of these chemical agents. There are several chemical substances with preservative properties that are used in cooked ham: sorbic acid (E-200), sodium benzoate (E-211), natamycin (E-235) or parahydroxybenzoates (E-218), which are still used in some countries (not in Spain). Meanwhile, sodium lactate, potassium lactate and sodium diacetate (E-325, E-326 and E-262, respectively) are used to a very limited extent or not at all in Spanish territory. It is important to mention the existence of plant extracts with antimicrobial functions that could allow the elimination of additives from the label. These drawbacks are related to the presence of allergens, the need to add bacteria with nitrate reductase activity, and the need to add vegetable flavourings and pigments [26]. There are several types of preservatives on the market, but the most widely used in Spain is undoubtedly sodium nitrite, which appears in practically all commercial brands of cooked ham. Nitrites (NO_2_^−^), and also nitrates (NO_3_^−^), are salts that have been used in the meat industry for many years, due to several functions: colour stabilisation, their inhibition of *Clostridium botulinum*, their contribution to the preservative effect of salt, and their contribution to the taste and aroma of the ham [26]. It is very important and necessary to control the amounts added, otherwise they can reach toxic levels. Therefore, in Regulation (EU) No. 1129/2011, the use of nitrates (E-251 and E-252) is not authorised in heat-treated meat products. In the case of nitrites, their use is limited to a maximum added amount of 150 ppm.
2. Antioxidants. Antioxidants are reducing substances that prevent the oxidation of other substances by oxidation–reduction reactions. The most used antioxidants in cooked ham are ascorbic acid/sodium ascorbate (E-300, E-301), its isomer isoascorbic acid, which is also called erythorbic acid/sodium erythorbate (E-315, E-316), sodium citrate (E-331) and sodium lactate (E-270). Sodium citrate and lactate play a reinforcing role, the former as a chelating and buffering agent and the latter as a depressant of water activity and inhibitor of lactobacilli [27,28]. The use of these reducing agents is permitted by European authorities in their acid form and in the salts they form with various cations [29,30]. Because of their greater prevalence in the meat industry, we will focus on ascorbic acid and its salts, as erythorbic acid and sodium erythorbate have the same functions [31]. Ascorbic acid is an organic compound that is highly soluble in water, but not in lipid media. In fact, its efficacy with respect to the inhibition of lipid oxidation is low, as reported by several authors [32,33]. Its use is frequent in the meat industry as it participates in the reduction of nitrite to nitric oxide, facilitating the formation of nitrosomyoglobin and, therefore, facilitating the stability of the pink colour in cooked ham [34]. It is also involved in preventing the appearance of nitrosamines, although the reaction mechanism has not been fully elucidated; however, it possibly blocks nitrosating agents [35,36]. It is recommended that it is added to the brine in its salt form and at an alkaline pH because otherwise, it may react with the nitrites and form irritating vapours.
3. Stabilisers. According to Real Decreto 142/2002, “stabilisers are substances that make it possible to maintain the physicochemical state of a foodstuff”. They include substances that allow the maintenance of a homogeneous dispersion of two or more immiscible substances in a food (emulsion), substances that stabilise, retain or intensify an existing colour in a food, and substances that increase the binding capacity of foods, including the formation of cross-links between proteins that allow the binding of food pieces In the reconstituted food product. The function of stabilisers in cooked ham is to reduce free water, improve or increase viscosity, and improve the functionality and physical stability of the product by providing firmness due to the reduction in or elimination of holes in the product. Among the most frequently used stabilisers in the cooked ham industry are locust bean gum, guar gum, xanthan gum, carrageenans, sorbitol or sorbitol syrup and phosphates (E-410, E-412, E-415, E-407, E-420 and E-451/452, respectively), the latter being the most present in practically all cooked ham put on sale.

The role of phosphates in cooked ham is to increase the WHC, preserve lipid oxidation, give stability to the emulsion, protect and stabilise the colour and also function as an antimicrobial. Its role is so fundamental that it can appear in low-salt products [37]. Phosphates are also involved in muscle binding due to their ability to alter the structure of myofibrillar proteins by having the capacity to dissociate actin and myosin. These chemical agents act on the long polypeptide chains that are found, forming tertiary and quaternary structures that are linked together by different types of bonds: hydrogen bridges, disulphide bridges and divalent cation bridges. Phosphates interfere with these electrostatic forces in a similar way to the sodium chloride, even more efficiently as they manage to open up the protein structure, increasing WHC. It is worth mentioning its chelating capacity, by which it traps divalent cations such as magnesium and calcium, and thus allows protein expansion [38]. Among the various types of phosphates, the ones that best perform the chelating function are the diphosphates (or pyrophosphates, P_2_O_4_^−^); however, they are rather insoluble in water and, therefore, it is necessary to use mixtures of pyrophosphate, tripolyphosphate and hexametaphosphate. Their antimicrobial capacity is related to their ability to chelate the bivalent ions (Nickel, Cobalt, Copper, Zinc) that are involved in the respiratory chain of bacteria and in their membranes’ structure [39]. Generally, it is not necessary to use concentrations that are higher than 5 g/kg of phosphate mixture in the brine. When these phosphates are added, the pH is altered. Alkaline phosphates increase the pH of the meat between 0.1 and 0.6 units, with pyrophosphate increasing it the most, followed by tripolyphosphate and hexametaphosphate [40]. According to European legislation, phosphates in any of their formulations (E-338-452) have a very limited use in some traditional or prepared meat products (Regulation (CE) No. 1099/2009).

The maximum amount of phosphates allowed by Spanish legislation is 5 g/kg, expressed as P_2_O_5_^−^ (Real Decreto 142/2002). Due to their use becoming increasingly restricted, it is necessary to look for alternatives in anticipation of a possible ban in the future. For this reason, several research groups have sought new ways in which the use of phosphates could be reduced or replaced with other healthier additives. The most common alternatives used to increase the WHC are as follows: starches (potato, rice), proteins (pea, carrot, beef), fibres, hydrocolloids and bicarbonate salts [41]. In the specific case of cooked ham, the group of Resconi et al. [25] tested rice starch and fructo-oligosaccharides as a substitute for phosphate and dextrose, respectively. The results showed that there was an improvement in WHC and yield after cooking, but it negatively affected its appearance and altered the ham’s organoleptic characteristics. Pancrazio et al. [42] tested the inclusion of beer yeast extract in cooked ham, reporting an increased firmness, chewiness and number of protein and free amino acids. Despite the interesting contributions that these additives can offer, it is important to remember that their inclusion can lead to the product having to change its denomination to include the term “fiambre”.

There are other processing methods that can help to replace or reduce phosphates, such as the application of ultrasound or high-pressure processing after brine injection. The use of ultrasound has been shown to promote brine diffusion into the meat matrix by halving the maturation period and reducing the amount of phosphates [43,44]. High-pressure processing leads to improved meat chewiness, toughness, better tenderness and reduced levels of cooking loss [45]. The application of high pressure onto cooked ham has been studied for its impact on the reduction and durability of *Serratia liquefaciens* [46]. Both mechanical strategies improve aspects of meat functionality but increase the cost of manufacturing cooked ham. Table 2 shows the ingredients and additives identified on the labelling of the different brands analysed.

## 6. Innovation and Development in Labelling

The labelling must contain relevant information about the type of product, its nutritional information and the presence of allergens in order to inform the consumer and prevent possible adverse effects. Labelling on products such as cooked ham is subject to European and Spanish regulations (Real Decreto 474/2014 and Regulation (EU) No. 1169/2011). There are a series of specifications that must appear on the labelling of cooked ham, and which are common to all brands in order to comply with the aforementioned legislation. In labelling, the name of the product is the most important part, as it defines the product itself. So that consumers can better identify and determine the different commercial categories of cooked ham, a circle with the following colours and dimensions must appear next to the name of the product: red for the extra cooked ham, green for the cooked ham and yellow for the “fiambre” of cooked ham.

Nutritional information is another set of important data. It should always be indicated in average values per 100 g; some brands choose to be even more specific and add the nutritional information per portion (~30–50 g), as well as the reference intake in order to calculate, in an indicative way, the amount of product that should be ingested to satisfy the amount of calories and nutrients that an adult needs on average; the units appear as a percentage. However, it is mandatory that the percentage of meat corresponds to that indicated on the labelling. Furthermore, any exogenous protein that has origin in a different animal or is of a different species (e.g., meat proteins, milk proteins, egg proteins, etc.) must appear on the labelling (Regulation (EU) No. 1169/2011).

The innovations that could be assessed in the labelling of cooked ham, according to the macro- and micro-nutrient composition, would be those relating to nutritional and health claims, based on Regulation (EC) No. 1924/2006 and Regulation (EU) No. 432/2012, as they could have a positive influence on the consumer’s purchasing decision. Table 3 shows the nutritional composition of the cooked ham, according to the Spanish information reference: this includes the Spanish Food Composition Database (BEDCA), the nutritional claims applicable, the conditions established in the legislation for their application (Regulations (EC) No. 1924/2006 and (EU) No. 1169/2011) and their fulfilment in cooked ham.

Based on its nutritional composition, cooked ham could include a wide range of nutritional claims, as well as some health claims, which are collected in the annex of Regulation (EU) No. 432/2012. Those claims are related to the bioactivity of proteins, vitamins and minerals that are found in a sufficient quantity for the ham to be considered as a “*source of*” them, i.e., thiamine, niacinamide, vitamin B12, iron, phosphorus, selenium and zinc. The average amount of fat does not exceed 3 g/100 g, so it would be in the limit for specifying the low-fat claim (<3 g fat/100 g). Nevertheless, it has some less desirable components such as sodium, although the current amounts added by producers do not exceed 2 g/100 g [2]. The consequence of including the positive nutritional claims is that any type of nutritional declaration should be also added. Hence, messages such as high sodium content should be included simultaneously (with the same characters type) when exceeding 700 mg/100 g, according to the levels indicated in the Working document on the setting of nutrient profiles [47].

## 7. Discussion

Innovation in a traditional and well-standardised product such as cooked ham is not easy, but it is a good opportunity to differentiate new products from other meat-producing companies. A great range of innovation options are possible in order to address the raw materials used, new potential ingredients and additives, the different stages of the production process, and even the sales format and ability to make attractive claims to consumers.

Traditionally, crossbreeds with low breeding costs have been used to produce cooked ham, but some companies are also turning to other breeds of pig that are more interesting from a technological and commercial point of view, such as Duroc. This breed presents higher levels of intramuscular fat than the other white breeds, which is related to juiciness in the final product and is highly appreciated by consumers.

To date, adequate substitutes that could be used to replace common additives in ham in order to advertise the product as “natural” have not yet been determined. In this vein, the most common improvements made have been in reducing the quantity of added salt and sugar, and finding alternatives in terms of stabilisers, which permit the utilization of the different chemical molecules of phosphates or others compounds, such as bean, guar, or xanthan gum, carrageenans, sorbitol, etc. Nevertheless, these advances have not being able to eliminate the most common antioxidants (as sodium erythorbate or ascorbate) or preservatives (sodium nitrite) used in order to maintain a good-quality product.

On the other hand, the introduction of changes into the production lines of cooked ham make the process easier, but it increases the production costs, thus opening up the possibility of offering the consumer products that are within the premium category. The most recommendable innovations in the industrial process of cooked ham that can be used to obtain a high-quality final product with an appropriate cost are related to the following: (i) the level of polishing of the raw material according to the quality or category of the final product; (ii) the amount of brine injected into the ham in order to obtain a final product with the ideal juiciness but with an appropriate percentage of leanness; (iii) the use of the optimum multi-needle system; (iv) the introduction of a maturation stage after the malaxing for a better brine distribution and improvements in the other organoleptic characteristics; (v) the setting of parameters (temperature and time) and choosing the correct cooking system, as it is essential to ensure that the correct pasteurisation and the adequate sensory experience of the product is achieved, since excessively high temperatures and time must be avoided in order to prevent the final product from being too hard and not very juicy due to the excessive loss of water. Finally, the product sale format is essential, as well as the information provided by the labelling, which needs to be attractive to the consumer. For that reason, nutritional and health claims should be considered for inclusion on labelling. In order to make a decision between the diverse cooked ham options, it would be necessary to evaluate the way that consumers perceive a particular claim considering its interest and attractiveness.

## Figures and Tables

**Table 1:** Categories and physicochemical characteristics of cooked ham.

Commercial Name	Commercial Category	Moisture/Protein Ratio	Collagen Free Protein	Total Soluble Sugars (g glucose/100 g)	Added Protein (g/100 g)	Starch (g glucose/100 g)
Cooked ham	Extra	≤4.13	-	≤1.5	Absence	Absence
Cooked ham			≥14.0	≤2.0	≤1.0	Absence
Fiambre				≤5.0	≤3.0	≤10.0

**Table 2:** Ingredients and additives of the different commercial brands of “extra” (E1–E4) and standard cooked ham (S1–S3).

Brand	Quantity of Meat (%)	Salt	Saccharose	Dextrose	Aroma	Stabilisers	Antioxidants	Preservatives
E1	90	X	X		X	E-407—Carrageenans E-508—Potassium chloride	E-326—Potassium lactate E301—Sodium ascorbate	E-250—Sodium nitrite
E2	90	X	X		X	E-407—Carrageenans E-451—Triphosphates		E-250—Sodium nitrite
E3	88	X	X		X	E-407—Carrageenans E-410—Xanthan gum E-415—Locust bean gum E-420—Sorbitols E-451i—Pentasodium triphosphate E-508—Potassium chloride	E-316—Sodium erythorbate	E-250—Sodium nitrite
E4	82	X	X		X	E-407—Carrageenans E-410—Xanthan gum E-415—Locust bean gum E-420—Sorbitols E-451i—Pentasodium triphosphate E-508—Potassium chloride	E-316—Sodium erythorbate	E-250—Sodium nitrite
S1	69	X	X		X	E-407—Carrageenans E-410—Xanthan gum E-415—Locust bean gum E-420—Sorbitols E-451i—Pentasodium triphosphate E-508—Potassium chloride	E-316—Sodium erythorbate	E-250—Sodium nitrite
S2	75	X		X		E-412—Guar gum E-450—Diphosphates E-451—Triphosphates E470a—Sodium, potassium and calcium salts of fatty acids	E-316—Sodium erythorbate	E-250—Sodium nitrite
S3	70	X	X		X	E-407—Carrageenans E-420—Sorbitols E-451—Triphosphates	E-316—Sodium erythorbate E-331—Sodium citrate	E-250—Sodium nitrite

X: means that it contains the additive/ingredient.

**Table 3:** Applicable nutritional claims in cooked ham.

	Per 100 g	Applicable Nutritional Claims	Terms of Use	Conditions in Cooked Ham
**Energy**, kcal	114			
**Water**, g	75.6			
**Proteins**, g	21	Source of proteins High in protein	≥12% energetic value ≥20% energetic value	energetic value of 84% from proteins
**Fats**, g	3	Low-fat	≤3 g fat/100 g	
Saturated fats	1.1	Low-saturated fat	Ʃsaturated fatty acids and trans-fatty acids ≤ 1.5 g/100 g Ʃsaturated fatty acids and trans-fatty ≤ 10% of energy	energetic value of 8.7% from saturated fats
Monounsaturated fats	1.4			
Polyunsaturated fats	0.36			
**Carbohydrates**, g	0.4	Sugar free	≤0.5 g per 100 g	
**Cholesterol**, mg	50			
**Vitamins**				
Thiamine (B1), mg	0.46	Source of thiamine High in thiamine	≥15% of NRV Twice value of “source of”	41.8% of NRV
Riboflavin (B2), mg	0.18			
Niacinamide equivalents (total) (B3), mg	11.4	Source of niacinamide High in niacinamide	≥15% of NRV Twice value of “source of”	71.3% of NRV
Pyridoxine (B6), mg	0.2			
Folic Acid (B9), µg	0.2			
Vitamin B12, µg	0.7	Source of vitamin B12	≥15% of NRV	28% of NRV
Vitamin C, mg	19			
Vitamin A, µg	Trace			
Vitamin D, µg	0.7			
Vitamin E, mg	0.08			
**Minerals**				
Calcium, mg	9.6			
Iron, mg	2.1	Source of iron	≥15% of NRV	15.1% of NRV
Potassium, mg	270			
Magnesium, mg	17.5			
Sodium, mg	970			
Phosphorus, mg	239	Source of phosphorous High in phosphorous	≥15% of NRV Twice value of “source of”	34.1% of NRV
Iodine, µg	10.9			
Selenium, µg	11	Source of selenium	≥15% of NRV	20% of NRV
Zinc, mg	2.8	Source of zinc	≥15% of NRV	28% of NRV

NVR: Nutrient Reference Value.

## Data Availability

Not applicable.
